# Transitional Care for Patients with Amyotrophic Lateral Sclerosis to the Home: A Scoping Review

**DOI:** 10.1590/0034-7167-2024-0297

**Published:** 2025-08-08

**Authors:** Evelyn Silva de Sousa, Larissa Arielly Cunha da Silva, Renilly de Melo Paiva, Katiane Domingos Soares, Isabelle Campos de Azevedo, Viviane Euzébia Pereira Santos

**Affiliations:** I Universidade Federal do Rio Grande do Norte. Natal, Rio Grande do Norte, Brazil

**Keywords:** Transitional Care, Domicile, Nursing, Amyotrophic Lateral Sclerosis, Health., Cuidado de Transición, Residencia, Enfermería, Esclerosis Amiotrófica Lateral, Salud.

## Abstract

**Objectives:**

to map the care required for the transition of patients with Amyotrophic Lateral Sclerosis to the home environment.

**Methods:**

a scoping review was conducted following the JBI guidelines. The search for publications on the topic was carried out in databases such as PubMed and Web of Science between February and May 2023. Full-text studies that addressed the research objective were included. Letters to the editor, editorials, and opinion articles were excluded.

**Results:**

the final sample consisted of seven articles. Regarding care, the studies highlighted assistance with basic life needs, care planning, protocol development, the use of telehealth, and communication between healthcare services.

**Conclusions:**

the care required for the transition of individuals with Amyotrophic Lateral Sclerosis to the home environment ranges from assistance with basic human needs to the coordination with other healthcare services. Altogether, these actions contribute to a safe and effective transitional process.

## RESUMEN

**Objetivos:**

mapear los cuidados necesarios para la transición del paciente con Esclerosis Lateral Amiotrófica al domicilio.

**Métodos:**

revisión de alcance conforme a las directrices del JBI. La búsqueda de publicaciones sobre el tema se realizó en bases de datos como *PubMed* y *Web of Science*, entre febrero y mayo de 2023. Se incluyeron estudios completos que respondían al objetivo de la investigación. Se excluyeron cartas al editor, editoriales y artículos de opinión.

**Resultados:**

la muestra final consistió en siete artículos. En cuanto a los cuidados, los estudios abordaron la asistencia a las necesidades básicas de vida, la planificación, la creación de protocolos, el uso de la telessaúde y la comunicación entre los servicios de salud.

**Conclusiones:**

los cuidados para la transición de la persona con Esclerosis Lateral Amiotrófica al domicilio van desde la asistencia a las necesidades humanas básicas hasta la coordinación con otros servicios de salud. Todo ello contribuye a un proceso de transición seguro y eficaz.

## INTRODUCTION

Amyotrophic Lateral Sclerosis (ALS) is a neurodegenerative disease that causes the progressive loss of motor neurons in the brain and spinal cord. Its etiology is multifactorial, with genetic and environmental associations. The initial clinical presentation is characterized by muscle weakness, loss of movement, and impaired motor coordination^(1)^.

Currently, ALS has no cure, and patient survival ranges from two to five years. In most cases, death occurs as a result of the most severe form of the disease, whose main consequence is ventilatory insufficiency caused by the failure of the affected respiratory muscles^(2)^. Therefore, the treatment of ALS patients aims to improve their quality of life throughout the progression of the disease.

To achieve this goal, it is necessary to combine pharmacological therapy with the continuous involvement of a multidisciplinary healthcare team. This process begins primarily with Primary Health Care (PHC), which investigates and addresses the initial symptoms of the disease, such as limb weakness and recurring cramps. However, more complex interventions are carried out in hospital settings, including mechanical ventilation (MV) and other procedures required to address ALS complications^(2,3)^.

Considering the patient holistically and acknowledging the prognosis of the disease, individuals with ALS at home require continuous care and a support network. This assistance is generally provided by a caregiver or family member, while the transition from hospital care to the home environment is facilitated by healthcare professionals. This dynamic fosters an integrated and longitudinal process of health promotion^(4)^.

Transitional care is, therefore, a set of actions aimed at ensuring the continuity of care across different settings or levels of healthcare, including the home environment^(5)^. In this context, strengthening the transfer of care from the hospital to the home represents an opportunity to provide care that is centered on the needs of the individual and their family. This approach promotes well-being, reduces hospital readmissions, minimizes complications and mortality, and lowers the costs associated with hospital admissions^(6)^.

To achieve this objective, it is essential that patients and their caregivers feel prepared and capable of implementing the care plans agreed upon during hospital discharge. To facilitate this process, the use of effective communication is indispensable. This goal aligns with the objectives set forth by the World Health Organization (WHO, 2021)^(7)^, which advocates for the strengthening of safe and effective health practices.

Moreover, it is essential that the multidisciplinary team responsible for this process identifies the patient’s needs, supports self-care, and develops a collaborative discharge plan. In this regard, it is crucial to identify the strengths and vulnerabilities of individuals with ALS, as well as to understand their health-disease process^(8)^.

It is important to highlight that, although the Care Transitions Measure^®^ (CTM), developed in the United States of America (USA), is widely used to assess the quality of care transitions^(9)^, most healthcare services still do not rely on specific instruments and protocols for transitional care. To consolidate this practice, it is imperative to train healthcare teams in the application of such instruments, with the goal of achieving a greater understanding and increased safety in the transition process.

In this context, considering that ALS is a disease with a reserved prognosis and a high impact on the quality of life of individuals, understanding the process of care transition for these patients allows for a more comprehensive understanding of the topic by healthcare professionals and family members involved^(8)^. Furthermore, there is a notable gap in the literature on transitional care, particularly in the context of ALS. This highlights the need for further exploration and deeper investigation into the subject.

Given this scenario, the following question arises: What are the care strategies for the transition of ALS patients to the home environment as addressed in scientific studies?

## OBJECTIVES

To map the care required for the transition of patients with ALS to the home environment.

## METHODS

### Ethical Aspects

Since this is a scoping review that exclusively utilizes publicly available data, review and approval by a Research Ethics Committee were not required.

### Type of Study

This study is a Scoping Review based on the guidelines of the JBI^(10)^ and conducted in accordance with the stages recommended by the international PRISMA-ScR^(11)^ framework. Prior to conducting this review, a research protocol was developed to guide the entire study process. The protocol is registered on the Open Science Framework (OSF) platform under the registration number osf.io/8pvbh. The registration was carried out prospectively, preceding the creation of the protocol.

The Scoping Review aims to synthesize information by mapping scientific studies published in national and international databases, with the objective of identifying concepts and information related to a specific area or topic. This approach facilitates the dissemination of scientific knowledge and addresses existing gaps in the literature^(12)^.

### Methodological Procedures

The structuring of this review was developed in the following stages: I - Identification of the research question, using the PCC strategy (Population, Concept, and Context); II - Identification of relevant studies; III - Selection of studies; IV - Data analysis; V - Synthesis and presentation of data; VI - Presentation of results.

To define the guiding question and the descriptors used for the search and selection of materials, the PCC strategy (Population, Concept, and Context) was applied. The terms used were drawn from the Descriptors in Health Sciences (DECS) and their English equivalents from the Medical Subject Headings (MeSH). Accordingly, the following elements were established: Population (P) - Patients with ALS / Patients with ALS; Concept (C) - Transitional Care / Transitional Care; Context (C) - Home Environment / Home Environment.

Based on this approach, the following search strategy was formulated: Amyotrophic Lateral Sclerosis OR (Motor neuron disease OR Neuromuscular disease) AND Transitional Care OR (Care transitions OR Continuity of care) AND Home Environment OR (Home healthcare services OR Home care).

Therefore, the guiding question for this review is as follows: “What are the care strategies for the transition of ALS patients to the home environment as addressed in scientific studies?”.

### Data Collection and Organization

A preliminary search was conducted to identify similar studies in national and international databases, as well as protocol records in the Open Science Framework (OSF). Since no relevant materials were found, access to the Cumulative Index to Nursing and Allied Health Literature (CINAHL) and U.S. National Library of Medicine (PubMed) databases was initiated to identify the most frequent and appropriate keywords for the study. In this context, with the help of the Boolean operators AND/E and OR/OU, the following search strategy was formulated: Amyotrophic Lateral Sclerosis OR (Motor neuron disease OR Neuromuscular disease) AND Transitional Care OR (Care transitions OR Continuity of care) AND Home Environment OR (Home healthcare services OR Home care).

The search for studies and their analysis was carried out from February to May 2023. The databases used were: U.S. National Library of Medicine (PubMed), Cumulative Index to Nursing and Allied Health Literature (CINAHL), Web of Science, Cochrane Library, PsycINFO, Latin American and Caribbean Health Sciences Literature (LILACS), SCOPUS, as well as searches in the CAPES Thesis and Dissertation Catalog, Education Resources Information Center (ERIC), The National Library of Australia’s Trove (TROVE), Academic Archive Online (DIVA), Europe E-Theses Portal (DART), Electronic Theses Online Service (EThOS), Open Access Scientific Repository of Portugal (RCAAP), and National ETD Portal.

Regarding the inclusion criteria, publications that addressed the study’s objective and were available in full through the CAPES Periodicals Portal, accessed via the Federated Academic Community (CAFe in Portuguese), were selected. This includes gray literature, such as theses and dissertations. Letters to the editor, editorials, and opinion articles were excluded. No restrictions were applied regarding the period of publication or the language in which the materials were produced. Additionally, no reference management software was used during the process.

### Data Analysis

The selection of articles was carried out through independent peer review. For this purpose, the following stages were followed: I - Screening of titles and abstracts of the findings; II - Full-text review of the studies pre-selected in the previous stage; III - Extraction of relevant information. In case of disagreement between the reviewers, a third reviewer was consulted.

For data synthesis, the following indicators were captured: databases, language, year of publication, country where the study was conducted, objective, methodological design, main transitional care practices for ALS patients, responsible party for this care, benefits and challenges encountered, and level of evidence, according to JBI^(9)^, as follows: Level 1 - Systematic review of randomized clinical trials;; Level 2 - Individual randomized clinical trial; Level 3.1 - Well-designed controlled clinical trials without randomization;; Level 3.2 - Well-designed cohort or case-control studies, analytical studies;; Level 3.3 - Multiple time series, with or without intervention, in uncontrolled experiments; Level 4 - Expert opinion.

At the end of the reading and analysis of the studies, the final sample was organized into a spreadsheet using Microsoft Excel 2019^®^, and subsequently presented in tables, graphs, and/or figures.

## RESULTS

**Figure 1:**
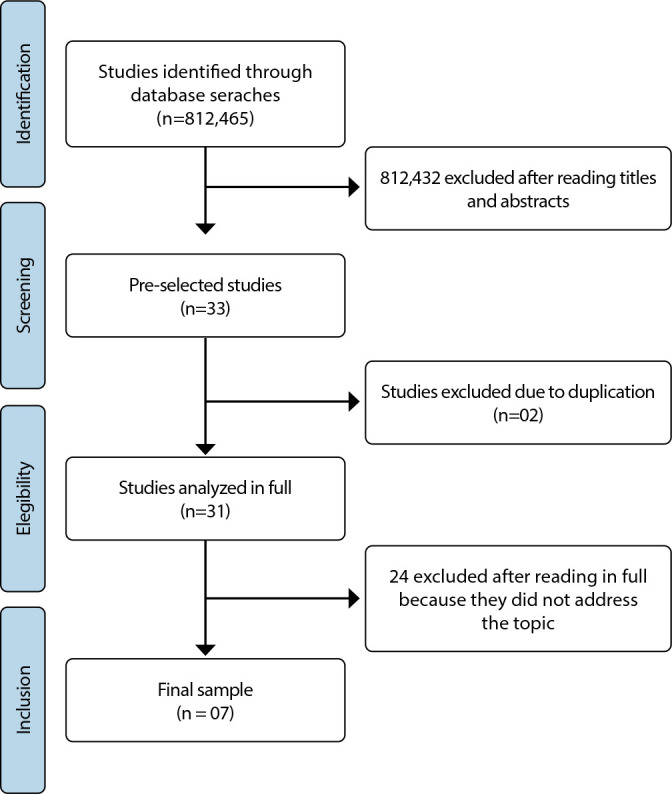
Flowchart of the stages of study selection, Natal, Rio Grande do Norte, Brazil, 2023

The search in the databases resulted in a total of 812,465 studies for analysis. During the initial screening, after evaluating the titles and abstracts, 33 studies were identified. After excluding two (2) duplicate articles, 31 studies were selected for full-text review. It is worth noting that the exclusion criteria for the articles were failure to address the research question and the duplication of identified studies. Ultimately, the final sample comprised seven (7) publications, all of which were articles (100%). Figure 1 illustrates the process of search, exclusion, and selection of the sample.

Regarding the year of publication, the included studies date from 2017 to 2023, with a notable concentration in 2022, which accounted for three publications (42.8%). In terms of the country of origin of the studies, Switzerland stood out with two studies (28.5%). The remaining studies presented a heterogeneous distribution, originating from Australia (1; 14.3%), Canada (1; 14.3%), France (1; 14.3%), the Netherlands (1; 14.3%), and Ireland (1; 14.3%).

With respect to the level of evidence of the studies, the majority were classified as level four (6; 85.7%), while only one article was classified as level 1 (1; 14.3%). This classification followed the parameters established by the Joanna Briggs Institute (JBI).

Additionally, it was identified that those responsible for providing transitional care were mostly members of the multidisciplinary healthcare team, such as physicians, nurses, and physical therapists, as well as informal caregivers.

Regarding the care provided to patients with ALS, the articles addressed a wide range of care activities, from those related to daily living activities, such as hygiene and rest, to care planning, protocol development, the use of telehealth, and communication between healthcare services (Chart 1). The selected studies were identified by the letter “A” followed by an ordinal number.

**Chart 1:** Care providers and key transitional care practices for patients with Amyotrophic Lateral Sclerosis (N=7), Natal, Rio Grande do Norte, Brazil, 2023

Reference^*^	Care Providers	Types of Care
Hodgen A; Foley G; Henderson RD; James N; Aoun SM, 2017^(13)^	Multidisciplinary team, including: physicians, physical therapists, nurses, social workers, and the neuropsychology team.	Counseling and support for the patient and family; Telehealth; Communication between healthcare services.
Poppe C; Schweikert K; Krones T, Wangmo T, 2022^(14)^	Nurses and informal home caregivers.	Advance care planning; Medical care coordination planning.
Lamy S; Veillard D; Doyen H; Kerbrat A; Michel L; Chretie E; et al, 2023^(15)^	Healthcare professionals in general.	Development of a standardized protocol for home administration of medication for ALS treatment.
Wu JM; Tam MT; Buch K; Khairati F; Wilson L; Bannerman E, 2022^(16)^	Informal caregivers.	Rest care, hygiene care (bathing).
Dontje ML; Reenen EK; Wijk E; Baars E; Visser-Meily JMA; Beelen A; et al, 2022^(17)^	Multidisciplinary healthcare teams for the treatment of individuals with ALS.	Use of telehealth to assist in the rehabilitation of ALS patients.
Poppe C; Verwey M; Wangmo T, 2021^(18)^	Informal caregivers.	Assistance with activities of daily living (hygiene, feeding); Support and counseling from specialized nurses; Psychological and spiritual support.
Galvin M; Carney S; Corr B; Mays I; Pender N; Hardiman O, 2018^(19)^	Informal caregivers.	Care planning.

*ALS - Amyotrophic Lateral Sclerosis.*

As shown in Chart 2, the sample also allowed for the identification of the main benefits that transitional care can provide to individuals with ALS, as well as the challenges faced by both patients and caregivers. Accordingly, three studies (42.85%) highlighted improvements in care organization and communication. Furthermore, regarding the challenges encountered, four studies (57.14%) emphasized stress and caregiver burden.

**Chart 2:** Benefits and challenges related to transitional care for patients with Amyotrophic Lateral Sclerosis (N=7), Natal, Rio Grande do Norte, Brazil, 2023

Reference^*^	Benefits	Difficulties
Hodgen A; Foley G; Henderson RD; James N; Aoun SM, 2017^(13)^	Use of telehealth for better monitoring of invasive and non-invasive ventilation at home; Improved communication between services.	Stress and burden for professionals dealing with the final stage of ALS; Lack of funding; insufficient preparation for professionals in care transitions and palliative care.
Poppe C; Schweikert K; Krones T, Wangmo T, 2022^(14)^	Not addressed.	Not addressed.
Lamy S; Veillard D; Doyen H; Kerbrat A; Michel L; Chretie E; et al, 2023^(15)^	Care organization.	Difficulty for patients in the technique of medication administration; Contradictory responses from healthcare professionals when doubts arose.
Wu JM; Tam MT; Buch K; Khairati F; Wilson L; Bannerman E, 2022^(16)^	Improvement of the emotional well-being of the individual with ALS.	Feelings of burden, stress, helplessness, physical overload.
Dontje ML; Reenen EK; Wijk E; Baars E; Visser-Meily JMA; Beelen A; et al, 2022^(17)^	The patient feels more in control of their care; easy communication with the care team; early identification of complications.	Slowness of the program used for telemedicine; non-specific questions and answers; lack of notification for new messages.
Poppe C; Verwey M; Wangmo T, 2021^(18)^	Training of informal caregivers; Development of plans for advanced care and end-of-life decisions.	Maintaining balance in the relationship with the individual with ALS; emotional and physical overload.
Galvin M; Carney S; Corr B; Mays I; Pender N; Hardiman O, 2018^(19)^	Better organization of daily care.	Need for caregiver supervision; lack of patient cooperation, behaviors, and resistance to external support, stress, and burden.

*ALS - Amyotrophic Lateral Sclerosis.*

## DISCUSSION

The results obtained allowed for the analysis and mapping of scientific evidence related to the care required for the transition of patients with ALS to the home environment. It is noteworthy that transitional care should be individualized, considering the specific needs of each person, especially those living with a neurodegenerative disease.

It was observed that studies on the topic had the highest prevalence in 2022. This finding may be related to the 74th World Health Assembly, which took place in 2021 and called for the creation of a Global Action Plan for Patient Safety^(7)^. The main objective of this plan is to achieve the maximum reduction of preventable harm in healthcare through the implementation of actions and tools that promote safe care. In this context, transitional care is an essential aspect, as it enhances healthcare assistance and contributes to the improved quality of life for patients^(7,20)^.

Regarding the origin of the publications, Europe stood out as the continent with the highest number of studies. This predominance may be associated with the high prevalence of ALS in Europe, which has an incidence rate of 10 to 12 cases per 100,000 people per year^(21)^. Among the European countries, Switzerland stood out, with two studies included in the sample. This may be related to the country’s high Human Development Index (HDI), which measures key social and economic factors relevant to the production of scientific knowledge and the implementation of more robust healthcare practices^(22)^.

In terms of levels of evidence, most studies (6; 85.7%) were classified as level 3 evidence, which includes non-experimental studies, such as qualitative research and case studies. This finding highlights the need for further investigations with higher levels of evidence to support the practical application of transitional care in the context of ALS patients. The scarcity of studies with higher levels of evidence may hinder the implementation of more robust evidence-based practices.

The multidisciplinary team was widely portrayed as the main party responsible for the transitional care of ALS patients to the home environment. These professionals possess fundamental skills and competencies to identify vulnerabilities and needs that arise during the transition from the hospital to the home. Furthermore, they provide the knowledge necessary for patients and their families to feel safe and capable of continuing care, thereby promoting greater involvement and autonomy^(8)^. In this sense, strengthening the competencies of the multidisciplinary team is a crucial aspect of ensuring the effectiveness of the transitional process.

With regard to the types of care that make up the transition, special emphasis was given to care planning^(14,19)^ and the use of telehealth^(13,17)^. Care management in the hospital environment is generally carried out during discharge planning. At this time, instructions are provided to the patient and family, support from other support networks is sought, and a detailed review of the patient’s clinical condition is conducted. However, a study conducted in Portugal^(23)^ identified gaps in discharge planning that compromise the effectiveness of the transition. Among the gaps highlighted were insufficient communication between healthcare professionals and the lack of standardization in care delivery. These factors compromise the continuity and safety of care, potentially resulting in hospital readmissions and other complications.

The findings of this review highlight the need to improve transitional care practices for home care in the context of ALS. The use of strategies such as effective planning, assertive communication, and strengthening the role of the multidisciplinary team are essential elements to ensure a safe and efficient transition. Finally, the importance of conducting new studies with greater methodological rigor and higher levels of evidence is emphasized to consolidate evidence-based practices and strengthen patient safety.

Telehealth is defined as the use of Information and Communication Technologies (ICT) to provide remote consultations and monitoring^(24)^. In the context of caring for individuals with ALS, the included studies^(13,17)^ demonstrated that this tool was useful for healthcare professionals to monitor medication use and the rehabilitation process of patients. Considering that ALS significantly impacts quality of life and is associated with polypharmacy - due to the multiplicity of symptoms that require management^(25)^ - the follow-up of these patients after hospitalization contributes to the monitoring and prevention of complications.

In Brazil, the Federal Nursing Council (COFEN in Portuguese), through Resolution 696/2022^(26)^, regulated the practices of tele-nursing and digital health. The objective of this regulation was to establish clear rules for the role of nursing professionals, as well as to ensure the provision of the necessary resources so that remote care occurs in a safe, controlled, and effective manner. It is worth noting that the practice of tele-nursing encompasses nursing consultations, remote monitoring, and health education, with all these activities being mediated by ICT.

Another relevant aspect of the transitional care of ALS patients to the home is the use of instruments and technologies that systematize the assistance process. An example widely cited in the literature is the CTM, which stands out as one of the most widely used instruments to assess the quality of care transitions in healthcare services^(9)^. The application of the CTM allows for an improvement in the quality of care, as technologies contribute to the safe and efficient transfer of care that was previously carried out exclusively in the hospital environment but, with the support of digital devices and tools, can now be carried out at home and/or in the community.

However, for transitional care to occur fully and effectively, it is essential to articulate the Health Care Networks (RAS), with special emphasis on PHC. This is because PHC is the setting where home visits, post-discharge consultations, health education actions, and the strengthening of the bond between the user and the health unit take place. In this scenario, the fragmentation of care is reduced, promoting benefits that range from continuous and progressive follow-up to the identification and resolution of emerging demands^(27)^.

Furthermore, the studies in the sample indicated that the organization of care and communication between services were fundamental elements for the success of the transition^(13,15,17,19)^. This was mainly due to the prior planning of care, which included the definition of strategies for patient follow-up at home.

Another point of note is the sense of protagonism and autonomy of individuals with ALS in their health-disease process^(17)^. Patient autonomy is a crucial element as it provides numerous benefits, such as increased adherence to treatment and a reduction in long-term costs. More participative and well-informed patients tend to use healthcare services more efficiently, thereby reducing the need for rehospitalization and other preventable complications^(28)^.

However, the process of transitioning ALS patients to home care also presents challenges. One of the main difficulties reported in the studies was the stress and burden experienced by family members, caregivers, and healthcare professionals. This is due to the fact that ALS is a highly complex condition with significant individual variability. As a result, healthcare teams, informal caregivers, and family members must be aware of the natural progression of the disease, its various clinical manifestations, and the psychological distress caused by a condition with no cure. In this context, providing emotional support, training, and coping strategies is essential to minimize the negative impacts on the mental health of those involved and to ensure comprehensive and humanized care.

These findings are consistent with a study^(29)^ that highlighted a moderate level of stress and burden among caregivers of patients with ALS. The main complaints reported by caregivers were related to the impact on their personal lives, such as reduced time for self-care, increased physical effort, and a sense of loss of freedom.

Given this scenario, it is possible to implement measures to mitigate these issues. A study^(30)^ that analyzed the burden on caregivers of people with ALS found that participation in discussion groups about the disease is beneficial. These groups serve as spaces for experience sharing, clarification of doubts, and the promotion of emotional support and qualified listening. This contributes to the emotional well-being of caregivers and improves their quality of life.

It is worth noting that care transition is still a sparsely discussed topic in Brazil. This gap is evident in the absence of programs or public policies that strengthen the practice of care transition at the national level. In contrast, countries like Canada have had guidelines since 2003 that support the continuity of care^(31)^. These Canadian guidelines serve as an international reference, as they promote coordination between different levels of care and ensure comprehensive and continuous patient assistance.

Therefore, it is essential to develop and implement public policies in Brazil that address care transition. Such policies can contribute to the autonomy of patients and caregivers, reduce the burden on those involved, and improve the continuity and quality of care, especially in the context of chronic and neurodegenerative diseases such as ALS.

### Study limitations

Among the limitations of this study, the low number of studies addressing the care required for the transition of patients with ALS stands out. This highlights the need for further investigations to facilitate a more comprehensive discussion on transitional care, especially in the context of neurodegenerative diseases. Future studies can contribute to the consolidation of evidence-based practices, thereby strengthening policies and strategies aimed at ensuring continuity of care.

### Contributions to the field of health and nursing

This research provides access to relevant knowledge on transitional care, a topic that is still underexplored, particularly with regard to ALS. The findings of this study can support the broader implementation of this process in healthcare services, as it qualifies care delivery and promotes the continuity and longitudinality of care. Additionally, the contributions presented here can serve as a basis for the formulation of public policies and the development of strategies that enhance the effectiveness of transitional care.

## CONCLUSIONS

This study enabled the identification of the main care practices involved in the transition of patients with ALS to the home environment. These practices include assistance with basic life needs, care planning, protocol development, the use of telehealth, and effective communication between healthcare services. Furthermore, it was found that this care is largely provided by a multidisciplinary team composed of professionals from various categories, ensuring the comprehensiveness of care.

The results obtained revealed significant benefits in care organization, improved communication between services, and the promotion of patient autonomy. Therefore, it is concluded that this study provides a relevant contribution to the scientific community and society in general by mapping the process of transitional care for ALS patients to the home environment.

Moreover, this work encourages the development of new investigations that can explore different contexts and scenarios, thereby strengthening the evidence supporting transitional care practices. It is hoped that healthcare professionals, managers, and civil society will foster broader discussions on this topic, enabling future collaborations and significant improvements in this process.

## Data Availability

Not applicable.
