# Supplementary material for: Transitional Care for Patients with Amyotrophic Lateral Sclerosis to the Home: A Scoping Review
Source: Rev Bras Enferm. 2025 Aug 8;78(3):e20240297. doi: 10.1590/0034-7167-2024-0297 (PMC12334203; doi:10.1590/0034-7167-2024-0297)
Supplement: Translation

# Translation: Cuidado Transicional do Paciente com Esclerose Lateral Amiotrófica para o Domicílio: Revisão de Escopo

**DOI:** 10.1590/0034-7167-2024-0297pt

**Authors:** Evelyn Silva de Sousa, Larissa Arielly Cunha da Silva, Renilly de Melo Paiva, Katiane Domingos Soares, Isabelle Campos de Azevedo, Viviane Euzébia Pereira Santos

**Affiliations:** I Universidade Federal do Rio Grande do Norte. Natal, Rio Grande do Norte, Brasil

**Keywords:** Cuidado Transicional, Domicílio, Enfermagem, Esclerose Lateral Amiotrófica, Saúde.

## Abstract

**Objetivos:**

mapear os cuidados para transição do paciente com Esclerose Lateral Amiotrófica para o domicílio.

**Métodos:**

revisão de escopo conforme as diretrizes JBI, as buscas de publicações sobre o tema ocorreram em bases como *PubMed* e *Web of Science*, entre fevereiro e maio de 2023. Foram incluídos estudos completos que respondiam ao objetivo da pesquisa. Cartas ao editor, editoriais e artigos de opinião foram excluídos.

**Resultados:**

a amostra final contou com sete artigos. Acerca dos cuidados, os estudos abordaram a assistência às necessidades básicas de vida, o planejamento, a criação de protocolos, o uso da telessaúde e a comunicação entre os serviços.

**Conclusões:**

os cuidados para transição do indivíduo com Esclerose Lateral Amiotrófica para o domicílio vão desde a assistência às necessidades humanas básicas até a articulação com outros serviços de saúde. Tudo isso, direciona para um processo transicional seguro e efetivo.

## INTRODUÇÃO

A Esclerose Lateral Amiotrófica (ELA) é uma doença neurodegenerativa que provoca a perda progressiva dos neurônios motores no cérebro e na medula espinhal. Sua etiologia é multifatorial, com associações genéticas e ambientais. O quadro clínico inicial caracteriza-se por fraqueza muscular, perda de movimento e de coordenação motora^(1)^.

Atualmente, a ELA não tem cura, e a sobrevida dos pacientes varia de dois a cinco anos. Na maioria dos casos, o óbito ocorre em decorrência da forma mais grave da doença, cuja principal consequência é a insuficiência ventilatória, ocasionada pela falha dos músculos respiratórios afetados^(2)^. Assim, o tratamento dos portadores de ELA tem como objetivo a melhora da qualidade de vida ao longo da progressão da doença.

Para isso, faz-se necessária a combinação da terapia medicamentosa com a atuação contínua da equipe multiprofissional de saúde. Esse processo inicia-se, principalmente, por meio da Atenção Primária à Saúde (APS), que investiga e atua nos sintomas iniciais da doença, como a fraqueza nos membros e as cãibras recorrentes. Entretanto, é no serviço hospitalar que as intervenções mais complexas são realizadas, incluindo a ventilação mecânica (VM) e outros procedimentos decorrentes das complicações da ELA^(2,3)^.

Desse modo, ao considerar o paciente de forma holística e o prognóstico da doença, o indivíduo com ELA, no domicílio, necessita de cuidados contínuos e de uma rede de apoio. Essa assistência é geralmente oferecida por um cuidador ou familiar, enquanto a transição do serviço hospitalar para o ambiente domiciliar é realizada pelos profissionais de saúde. Essa dinâmica favorece um processo integral e longitudinal de promoção à saúde^(4)^.

O cuidado transicional é, portanto, um conjunto de ações que visam à continuidade da assistência entre diferentes locais ou níveis de atenção à saúde, incluindo o domicílio^(5)^. Nesse sentido, fortalecer a transferência da assistência do ambiente hospitalar para a residência representa uma oportunidade de oferecer um cuidado centrado nas necessidades da pessoa e de sua família, promovendo bem-estar, reduzindo reinternações, complicações, mortalidade e custos com internações hospitalares^(6)^.

Para alcançar esse objetivo, é essencial que o paciente e seus cuidadores se sintam preparados e aptos a implementar os planos assistenciais acordados durante a alta hospitalar. Para que isso ocorra, torna-se indispensável a utilização de uma comunicação efetiva, que faz parte dos objetivos preconizados pela *World Health Organization* (WHO, 2021)^(7)^. A organização propõe o fortalecimento de práticas de saúde seguras e eficazes.

Outrossim, é fundamental que a equipe multiprofissional responsável por esse processo observe as demandas do paciente, apoie o autocuidado e elabore um planejamento de alta colaborativo. Nesse sentido, é necessário identificar as potencialidades e fragilidades do indivíduo com ELA, bem como o seu processo saúde-doença^(8)^.

É importante ressaltar que, embora exista o *Care Transitions Measure^®^* (CTM), desenvolvido nos Estados Unidos da América (EUA) e amplamente utilizado para avaliar a qualidade da transição do cuidado^(9)^, a maioria dos serviços de saúde ainda não se baseia em instrumentos e protocolos específicos para o cuidado transicional. Para que essa prática seja consolidada, é imprescindível o treinamento das equipes de saúde na aplicação de tais instrumentos, visando a obtenção de maior entendimento e segurança no processo de transição.

Nesse contexto, considerando que a ELA é uma doença de prognóstico reservado e de alto impacto na qualidade de vida dos indivíduos, conhecer o processo de transição do cuidado desses pacientes permite uma maior compreensão da temática por parte dos profissionais de saúde e dos familiares envolvidos^(8)^. Ademais, observa-se uma lacuna na literatura sobre o cuidado transicional, especialmente no contexto da ELA. Isso reforça a necessidade de busca e aprofundamento de estudos que abordem a temática.

Diante desse cenário, questiona-se: quais os cuidados para a transição do paciente com ELA para o domicílio que são abordados nos estudos científicos?

## OBJETIVOS

Mapear os cuidados necessários para a transição do paciente com ELA para o domicílio.

## MÉTODOS

### Aspectos éticos

Por se tratar de uma revisão de escopo que utiliza exclusivamente dados de domínio público, não foi necessária a apreciação pelo Comitê de Ética em Pesquisa.

### Tipo de Estudo

Trata-se de uma Revisão de Escopo baseada nas instruções do JBI^(10)^ e conduzida conforme as etapas recomendadas pelo guia internacional *PRISMA-ScR* ^(11)^. Previamente à realização desta revisão, foi elaborado um protocolo de pesquisa que orientou todo o processo do estudo. O protocolo encontra-se registrado na plataforma *Open Science Framework* (OSF), sob o número osf.io/8pvbh. O registro foi realizado de forma prospectiva, antecedendo a criação do protocolo.

A Revisão de Escopo promove uma síntese de informações por meio do levantamento de estudos científicos publicados em bases de dados nacionais e internacionais, com o objetivo de identificar conceitos e informações sobre uma determinada área ou temática. Essa abordagem possibilita a disseminação do conhecimento científico e o preenchimento de lacunas existentes na literatura^(12)^.

### Procedimentos Metodológicos

A estruturação desta revisão foi desenvolvida em etapas: I - Identificação da questão de pesquisa, em que se usou a estratégia PCC (População, Conceito e Contexto); II - Identificação dos estudos relevantes; III - Seleção dos estudos; IV- Análise dos dados; V - Síntese e apresentação dos dados e VI - Apresentação dos resultados.

Para definição da questão norteadora e dos descritores para busca e seleção dos materiais, foi utilizada a estratégia PCC (População, Conceito e Contexto), em que os termos empregados estão nos Descritores em Ciências da Saúde (DECS) e seus equivalentes em inglês no *Medical Subject Headings* (MESH). Logo, tem-se População (P)- Pacientes com ELA/ Patients with ALS; Conceito (C)- Cuidado Transicional/ Transitional Care; Contexto (C)- Ambiente Domiciliar/Home Environment. Nessa conjuntura, obteve-se a seguinte estratégia de busca: *Amyotrophic Lateral Sclerosis OR (Motor neuron disease OR Neuromuscular disease) AND Transitional Care OR (Care transitions OR Continuity of care) AND Home Environment OR (Home healthcare services OR Home care).*

Assim, tem-se a seguinte questão norteadora: “Quais os cuidados para a transição do paciente com ELA para o domicílio abordados nos estudos científicos?”.

### Coleta e organização dos dados

Realizou-se uma busca prévia para identificar estudos semelhantes em bases de dados nacionais e internacionais, bem como registros de protocolos no *Open Science Framework* (OSF). Como não foram encontrados materiais relevantes, deu-se início ao acesso às bases de dados *Cumulative Index to Nursing and Allied Health Literature* (CINAHL) e *U.S. National Library of Medicine* (PubMed) para identificar as palavras-chave mais frequentes e adequadas ao estudo. Nessa conjuntura, com o auxílio dos operadores booleanos AND/E e OR/OU, formulou-se a seguinte estratégia de busca: *Amyotrophic Lateral Sclerosis* OR (*Motor neuron disease* OR *Neuromuscular disease*) AND *Transitional Care* OR (*Care transitions* OR *Continuity of care*) AND *Home Environment* OR (*Home healthcare services* OR *Home care*).

A busca pelos estudos e suas análises foi realizada no período de fevereiro a maio de 2023. As bases de dados utilizadas foram: *U.S. National Library of Medicine* (PubMed), *Cumulative Index to Nursing and Allied Health Literature* (CINAHL), *Web of Science, Biblioteca Cochrane, PsycINFO, Literatura Latino-Americana e do Caribe em Ciências da Saúde* (LILACS), *SCOPUS*, além de buscas no *Catálogo de Teses e Dissertações da CAPES, Education Resources Information Center* (ERIC), *The National Library of Australia’s Trove* (TROVE), *Academic Archive Online* (DIVA), *Europe E-Theses Portal* (DART), *Electronic Theses Online Service* (EThOS), *Repositório Científico de Acesso Aberto de Portugal* (RCAAP) e *National ETD Portal*.

Em relação aos critérios de inclusão, foram selecionadas publicações que respondessem ao objetivo do estudo e que estivessem disponíveis na íntegra por meio do Portal de Periódicos CAPES, acessado pela Comunidade Acadêmica Federada (café), o que inclui a literatura cinzenta, como teses e dissertações. Excluíram-se cartas ao leitor, editoriais e artigos de opinião. Não se delimitou o período de publicação nem a língua em que os materiais foram produzidos. Ademais, não se utilizou gerenciador de referências durante o processo.

### Análise dos dados

A seleção dos artigos, portanto, ocorreu mediante revisão por pares, de maneira independente. Para isso, seguiu-se as seguintes etapas: I - Leitura dos títulos e resumos dos achados; II - Leitura na íntegra dos trabalhos pré-selecionados na etapa anterior e III- Extração das informações pertinentes. Em caso de incompatibilidade entre os revisores um terceiro era consultado.

Para a síntese dos dados, capturou-se os seguintes indicadores: bases de dados, idioma, ano de publicação, país onde o estudo foi realizado, objetivo, desenho metodológico, principais cuidados de transição ao paciente com ELA, responsável por esse cuidado, seus benefícios e dificuldades encontradas e nível de evidência, conforme o JBI^(9)^, sendo: Nível 1 - Revisão sistemática de ensaios clínicos randomizados; Nível 2 - Ensaio clínico randomizado individual; Nível 3.1 - Ensaios clínicos controlados bem delineados, sem randomização; Nível 3.2 - Estudos de coorte bem delineados ou caso-controle, estudos analíticos; Nível 3.3 - Séries temporais múltiplas, com ou sem intervenção em experimentos não controlados; Nível 4 - Parecer de especialistas.

Ao final da leitura e análise dos trabalhos, a amostra final foi organizada em planilha por meio do software *Microsoft Excel* 2019^®^, em seguida, foi disposta e apresentada em tabelas, gráficos e/ou figuras.

## RESULTADOS

A busca nas bases de dados resultou em um total de 812.465 estudos para análise. Na triagem inicial, ao avaliar título e resumo, obteve-se 33 trabalhos, após a exclusão de dois (2) artigos duplicados, obteve-se 31 para leitura na íntegra. Realça-se que os critérios para exclusão dos artigos foram não atender a questão de pesquisa, além da duplicidade de trabalhos encontrados. Por fim, compuseram a amostra final sete (7) publicações, das quais todas são artigos (100%). A Figura 1 demonstra o processo de busca, exclusão e seleção da amostra encontrada.

**Figura 1:** Fluxograma das etapas de seleção dos estudos, Natal, Rio Grande do Norte, Brasil, 2023

Acerca do ano de publicação, os trabalhos incluídos datam de 2017 a 2023, com destaque para o ano de 2022, que concentrou três publicações (42,8%). No que se refere ao país de origem dos estudos, a Suíça se sobressaiu, com dois estudos (28,5%). Os demais apresentaram uma distribuição heterogênea, sendo provenientes da Austrália (1; 14,3%), Canadá (1; 14,3%), França (1; 14,3%), Holanda (1; 14,3%) e Irlanda (1; 14,3%).

Quanto ao nível de evidência dos estudos, a maioria foi classificada como nível quatro (6; 85,7%), enquanto apenas um artigo foi de nível 1 (1; 14,3%). Para essa classificação, utilizaram-se os parâmetros estipulados pelo *Joanna Briggs Institute* (JBI).

Além disso, identificou-se que os responsáveis pelos cuidados de transição são, em sua maioria, membros da equipe multiprofissional de saúde, como médicos, enfermeiros e fisioterapeutas, além de cuidadores informais.

No que diz respeito aos cuidados ao paciente com ELA, os artigos abordaram desde aqueles relacionados às atividades de vida diária, como higiene e repouso, até o planejamento dos cuidados, a criação de protocolos, o uso da telessaúde e a comunicação entre os serviços de saúde (Quadro 1).

**Quadro 1:** Responsáveis pelo cuidado prestado e principais cuidados para transição do paciente com Esclerose Lateral Amiotrófica (N=7), Natal, Rio Grande do Norte, Brasil, 2023

Estudos^*^	Responsáveis pelo cuidado	Tipos de Cuidados
Hodgen A; Foley G; Henderson RD; James N; Aoun SM, 2017^(13)^	Equipe multiprofissional dentre os quais: médicos, fisioterapeutas, enfermeiros, serviço social e equipe de neuropsicologia.	Aconselhamento e apoio ao paciente e familiar; Telessaúde; Comunicação entre os serviços de saúde.
Poppe C; Schweikert K; Krones T, Wangmo T, 2022^(14)^	Enfermeiros e cuidadores domiciliares informais.	Planejamento antecipado dos cuidados; Planejamento de coordenação de cuidados médicos.
Lamy S; Veillard D; Doyen H; Kerbrat A; Michel L; Chretie E; et al, 2023^(15)^	Profissionais de saúde em geral.	Criação de um protocolo padronizado para administração domiciliar de medicamento destinado ao tratamento da ELA.
Wu JM; Tam MT; Buch K; Khairati F; Wilson L; Bannerman E, 2022^(16)^	Cuidadores informais.	Cuidados de repouso, cuidados de higiene (banho).
Dontje ML; Reenen EK; Wijk E; Baars E; Visser-Meily JMA; Beelen A; et al, 2022^(17)^	Equipes de saúde multidisciplinares de tratamento à pessoa com ELA.	Uso de telessaúde para auxiliar na reabilitação dos pacientes com ELA.
Poppe C; Verwey M; Wangmo T, 2021^(18)^	Cuidadores informais.	Auxílio em atividades de vida diária (higiene, alimentação); Apoio e aconselhamento de enfermeiras especializadas; Apoio psicológico e espiritual.
Galvin M; Carney S; Corr B; Mays I; Pender N; Hardiman O, 2018^(19)^	Cuidadores informais.	Planejamento do cuidado.

*ELA - Esclerose Lateral Amiotrófica.*

Como exposto no Quadro 2, a amostra, também, permitiu apontar os principais benefícios que os cuidados de transição podem proporcionar ao indivíduo com ELA, como também as dificuldades encontradas pelo paciente e cuidador. Assim, três trabalhos (42,85%) se relacionaram com a melhora na organização do cuidado e comunicação. Outrossim, no que tange as dificuldades encontradas, quatro estudos (57,14%) destacaram o estresse e a sobrecarga.

**Quadro 2:** Benefícios e dificuldades relacionados aos cuidados para transição do paciente com Esclerose Lateral Amiotrófica (N=7), Natal, Rio Grande do Norte, Brasil, 2023

Estudos^*^	Benefícios	Dificuldades
Hodgen A; Foley G; Henderson RD; James N; Aoun SM, 2017^(13)^	Uso da telessaúde para melhor monitorização da ventilação invasiva e não invasiva no domicílio; Maior comunicação entre os serviços.	Estresse e sobrecarga para os profissionais que lidam com a fase final da ELA; Falta de financiamento; pouca preparação para os profissionais na transição do cuidado e cuidados paliativos.
Poppe C; Schweikert K; Krones T, Wangmo T, 2022^(14)^	Não aborda.	Não aborda.
Lamy S; Veillard D; Doyen H; Kerbrat A; Michel L; Chretie E; et al, 2023^(15)^	Organização do cuidado.	Dificuldade dos pacientes na técnica de administração do medicamento; Respostas contraditórias por parte dos profissionais de saúde quando dúvidas surgiam.
Wu JM; Tam MT; Buch K; Khairati F; Wilson L; Bannerman E, 2022^(16)^	Melhora do bem-estar emocional do indivíduo com ELA.	Sentimentos de sobrecarga, estresse, desamparo, sobrecarga física.
Dontje ML; Reenen EK; Wijk E; Baars E; Visser-Meily JMA; Beelen A; et al, 2022^(17)^	Paciente se sente mais no controle do seu cuidado; fácil comunicação com a equipe de cuidados; identificação precoce de agravos.	Lentidão do programa usado para a telemedicina; perguntas e respostas não específicas; falta de notificação de novas mensagens.
Poppe C; Verwey M; Wangmo T, 2021^(18)^	Capacitação dos cuidadores informais; Criação de planos para cuidados avançados e decisões de fim de vida.	Manter o equilíbrio no relacionamento com o indivíduo com ELA; sobrecarga emocional e física.
Galvin M; Carney S; Corr B; Mays I; Pender N; Hardiman O, 2018^(19)^	Maior organização do cuidado diário.	Necessidade de supervisão dos cuidadores; falta de cooperação dos pacientes, comportamentos e resistência ao suporte externo, estresse e sobrecarga.

*ELA - Esclerose Lateral Amiotrófica.*

## DISCUSSÃO

Os resultados obtidos permitiram a análise e o mapeamento das evidências científicas relacionadas aos cuidados necessários para a transição do paciente com ELA para o domicílio. Destaca-se que a transição do cuidado deve ser individualizada, considerando as necessidades específicas de cada pessoa, especialmente aquelas que convivem com uma doença neurodegenerativa.

Observou-se que os estudos sobre a temática apresentaram maior prevalência no ano de 2022. Esse fato pode estar relacionado ao advento da 74ª Assembleia Mundial da Saúde, ocorrida em 2021, que determinou a criação de um Plano de Ação Global para Segurança do Paciente^(7)^. O referido plano tem como objetivo principal a máxima redução de danos evitáveis nos cuidados de saúde, por meio da implementação de ações e ferramentas que promovam o cuidado seguro. Nesse contexto, a transição do cuidado é um aspecto essencial, pois qualifica a assistência e contribui para a melhora da qualidade de vida dos pacientes^(7,20)^.

Quanto à origem das publicações, a Europa destacou-se como o continente com o maior número de estudos. Essa predominância pode estar associada à alta prevalência da ELA no continente europeu, cuja taxa é de 10 a 12 casos por 100.000 pessoas por ano^(21)^. Dentre os países europeus, a Suíça sobressaiu, com dois estudos incluídos na amostra. Esse fator pode estar relacionado ao elevado Índice de Desenvolvimento Humano (IDH) do país, que mede fatores sociais e econômicos relevantes para a produção de conhecimento científico e a implementação de práticas de saúde mais robustas^(22)^.

Em relação aos níveis de evidência dos estudos, a maioria (6; 85,7%) foi classificada como nível 3 de evidência, o que inclui estudos não experimentais, como pesquisas qualitativas e estudos de caso. Esse achado sinaliza a necessidade de novas investigações com níveis de evidência mais elevados, de forma a subsidiar a aplicação prática dos cuidados transicionais no contexto do paciente com ELA. A escassez de estudos com níveis de evidência superiores pode limitar a implementação de práticas baseadas em evidências mais robustas.

A equipe multiprofissional foi amplamente retratada como a principal responsável pelo cuidado transicional do paciente com ELA para o domicílio. Esses profissionais possuem competências e habilidades fundamentais para identificar fragilidades e necessidades que surgem durante a transição do ambiente hospitalar para a residência. Além disso, eles fornecem o conhecimento necessário para que o paciente e sua família se sintam seguros e aptos a dar continuidade ao cuidado, promovendo maior envolvimento e autonomia^(8)^. Nesse sentido, o fortalecimento das competências da equipe multiprofissional é um aspecto crucial para a efetividade do processo transicional.

Quanto aos tipos de cuidados que integram a transição, houve destaque para o planejamento dos cuidados^(14,19)^ e para o uso da telessaúde^(13,17)^. O gerenciamento dos cuidados no ambiente hospitalar é geralmente realizado no decorrer do planejamento da alta hospitalar. Nesse momento, são fornecidas instruções ao paciente e à família, busca-se apoio de outras redes de suporte, além de se realizar uma revisão detalhada do quadro clínico do paciente. No entanto, uma pesquisa realizada em Portugal^(23)^ evidenciou lacunas no planejamento da alta, as quais comprometem a efetividade da transição. Entre as lacunas apontadas, destacam-se a comunicação insuficiente entre os profissionais de saúde e a ausência de padronização na assistência. Esses fatores comprometem a continuidade e a segurança do cuidado, podendo resultar em readmissões hospitalares e outras complicações.

Os achados desta revisão indicam a necessidade de aprimoramento das práticas de transição do cuidado para o domicílio no contexto da ELA. A utilização de estratégias como o planejamento eficaz, a comunicação assertiva e o fortalecimento do papel da equipe multiprofissional são elementos essenciais para assegurar uma transição segura e eficiente. Por fim, destaca-se a importância de realizar novas pesquisas de maior rigor metodológico, com níveis de evidência superiores, a fim de consolidar as práticas baseadas em evidências e fortalecer a segurança do paciente.

A telessaúde é definida como o uso de Tecnologias da Informação e Comunicação (TIC) para oferecer consultas e monitoramentos a distância^(24)^. No contexto do cuidado ao indivíduo com ELA, os estudos incluídos^(13,17)^ evidenciaram que essa ferramenta foi útil para que os profissionais de saúde pudessem acompanhar o uso de medicamentos e o processo de reabilitação dos pacientes. Considerando que a ELA compromete significativamente a qualidade de vida e está associada à polifarmácia - devido à multiplicidade de sintomas que necessitam de controle^(25)^ -, o acompanhamento desses pacientes após a hospitalização contribui para a vigilância e a prevenção de complicações.

No Brasil, o Conselho Federal de Enfermagem (COFEN), por meio da Resolução 696/2022^(26)^, normatizou as práticas de telenfermagem e saúde digital. O objetivo dessa regulamentação foi estabelecer regras claras para a atuação dos profissionais de enfermagem, além de garantir a oferta de insumos necessários para que o atendimento remoto ocorra de forma segura, controlada e resolutiva. Ressalta-se que a prática da telenfermagem abrange consultas de enfermagem, monitoramento remoto e a própria educação em saúde, sendo todas essas atividades mediadas pelas TIC.

Outro aspecto relevante para o cuidado transicional do paciente com ELA para o domicílio é a utilização de instrumentos e tecnologias que sistematizem o processo de assistência. Um exemplo amplamente citado na literatura é o CTM, que se destaca como um dos instrumentos mais utilizados para avaliar a qualidade da transição do cuidado nos serviços de saúde^(9)^. A aplicação do CTM permite a elevação da qualidade da assistência, uma vez que as tecnologias contribuem para a transferência segura e eficiente de cuidados que, anteriormente, eram realizados exclusivamente no ambiente hospitalar, mas que, com o apoio de dispositivos e ferramentas digitais, passam a ser realizados no domicílio e/ou na comunidade.

No entanto, para que o cuidado transicional ocorra de forma plena e efetiva, é fundamental a articulação entre as Redes de Atenção à Saúde (RAS), com destaque para a APS. Isso porque a APS é o espaço onde ocorrem visitas domiciliares, consultas pós-alta, ações de educação em saúde e o fortalecimento do vínculo entre o usuário e a unidade de saúde. Nesse cenário, a fragmentação da assistência se reduz, promovendo benefícios que vão desde o acompanhamento contínuo e progressivo até a identificação e resolução de demandas emergentes^(27)^.

Ademais, os estudos da amostra apontaram que a organização dos cuidados e a comunicação entre os serviços foram elementos fundamentais para o sucesso da transição^(13,15,17,19)^. Isso ocorreu, sobretudo, devido ao planejamento prévio dos cuidados, que incluiu a definição de estratégias para o acompanhamento do paciente no domicílio.

Outro ponto de destaque é a sensação de protagonismo e autonomia do indivíduo com ELA no seu processo saúde-doença^(17)^. A autonomia do paciente é um elemento crucial, pois proporciona inúmeros benefícios, como a maior adesão ao tratamento e a redução de custos a longo prazo. Pacientes mais participativos e bem informados tendem a utilizar os serviços de saúde de forma mais eficiente, reduzindo a necessidade de reinternações e outras complicações evitáveis^(28)^.

Contudo, o processo de transição do paciente com ELA para o domicílio também apresenta desafios. Uma das principais dificuldades relatadas nos estudos foi o estresse e a sobrecarga vivenciados por familiares, cuidadores e profissionais de saúde. Isso se deve ao fato de que a ELA é uma condição de alta complexidade e com grande variabilidade individual. Dessa forma, a equipe de saúde, os cuidadores informais e os familiares precisam estar cientes da progressão natural da doença, das diferentes manifestações clínicas e do sofrimento psíquico causado por uma enfermidade sem cura. Nesse contexto, oferecer apoio emocional, treinamento e estratégias de enfrentamento se faz essencial para minimizar os impactos negativos na saúde mental dos envolvidos e garantir uma assistência integral e humanizada.

Esses achados estão em consonância com o estudo^(29)^ que evidenciou uma sobrecarga moderada de estresse em cuidadores de pacientes com ELA. As principais queixas relatadas pelos cuidadores estão relacionadas a implicações na vida pessoal, como a diminuição do tempo disponível para o autocuidado, o aumento do esforço físico e a sensação de perda de liberdade.

Diante desse cenário, é possível implementar medidas que atenuem essas problemáticas. Uma pesquisa^(30)^ que analisou a sobrecarga de cuidadores de pessoas com ELA verificou que a participação em grupos de discussão sobre a doença é benéfica. Esses grupos funcionam como espaços de troca de experiências, esclarecimento de dúvidas e promoção do acolhimento e da escuta qualificada, o que contribui para o fortalecimento emocional dos cuidadores e para a melhoria de sua qualidade de vida.

Ressalta-se que a transição do cuidado ainda é um tema pouco debatido no Brasil. Essa lacuna é perceptível pela ausência de programas ou políticas públicas que fortaleçam a prática da transição no território nacional. Em contrapartida, países como o Canadá possuem, desde 2003, diretrizes que subsidiam a continuidade do cuidado^(31)^. Essas diretrizes canadenses servem de referência internacional, uma vez que promovem a articulação entre os diferentes níveis de atenção e garantem a assistência integral e contínua ao paciente.

Assim, reforça-se a importância de se desenvolver e implementar políticas públicas no Brasil que abordem a transição do cuidado. Essas políticas podem contribuir para a autonomia dos pacientes e cuidadores, reduzir a sobrecarga dos envolvidos e melhorar a continuidade e a qualidade da assistência, especialmente no contexto de doenças crônicas e neurodegenerativas, como a ELA.

### Limitações do estudo

Dentre as limitações deste estudo, destaca-se o baixo quantitativo de trabalhos que abordam os cuidados necessários para a transição do paciente com ELA. Esse fato reflete a necessidade de novas investigações que possibilitem um debate mais abrangente sobre o cuidado transicional, especialmente no contexto de doenças neurodegenerativas. Estudos futuros podem contribuir para a consolidação de práticas baseadas em evidências, fortalecendo as políticas e estratégias voltadas para a continuidade do cuidado.

### Contribuições para a área da saúde e da enfermagem

Esta pesquisa proporciona o acesso a um conhecimento relevante acerca do cuidado transicional, uma temática ainda pouco explorada, especialmente no que se refere à ELA. Os achados deste estudo podem subsidiar a implementação desse processo de forma mais ampla nos serviços de saúde, uma vez que ele qualifica a assistência e promove a continuidade e a longitudinalidade do cuidado. Além disso, as contribuições aqui apresentadas podem servir de base para a formulação de políticas públicas e a criação de estratégias que promovam a efetividade do cuidado transicional.

## CONCLUSÕES

Este estudo permitiu identificar os principais cuidados envolvidos na transição do paciente com ELA para o domicílio. Entre eles, destacam-se a assistência às necessidades básicas de vida, o planejamento de cuidados, a criação de protocolos, o uso da telessaúde e a comunicação eficaz entre os serviços de saúde. Ademais, constatou-se que esses cuidados, em grande parte, são realizados por uma equipe multiprofissional, composta por profissionais de diferentes categorias, o que garante a integralidade da assistência.

Os resultados obtidos apontaram benefícios significativos na organização do cuidado, na melhoria da comunicação entre os serviços e na promoção da autonomia do paciente. Dessa forma, depreende-se que este estudo oferece uma contribuição relevante para a comunidade científica e para a sociedade em geral, ao mapear o processo de transição do cuidado do paciente com ELA para o domicílio.

Além disso, este trabalho incentiva a realização de novas investigações que possam explorar diferentes contextos e cenários, contribuindo para o fortalecimento de evidências que sustentem as práticas de cuidado transicional. Assim, espera-se que os profissionais de saúde, os gestores e a própria sociedade civil promovam discussões mais amplas sobre a temática, a fim de que futuras articulações e melhorias significativas sejam efetivadas nesse processo.

## Data Availability

Não se aplica.
